# Inferring regulatory element landscapes and gene regulatory networks from integrated analysis in eight hulless barley varieties under abiotic stress

**DOI:** 10.1186/s12864-022-09070-x

**Published:** 2022-12-20

**Authors:** Qijun Xu, Shunmou Huang, Ganggang Guo, Chunbao Yang, Mu Wang, Xingquan Zeng, Yulin Wang

**Affiliations:** 1 State Key Laboratory of Hulless Barley and Yak Germplasm Resources and Genetic Improvement, Lhasa, 850002 China; 2 Agricultural Research Institute, Tibet Academy of Agricultural and Animal Husbandry Sciences, Lhasa, 850002 China; 3 College of Forestry, Henan Agricultural University, Zhengzhou, 450002 People’s Republic of China; 4 Institute of Crop Sciences, Chinese Academy of Agricultural Sciences, Beijing, 100081 China

**Keywords:** BS-seq, ChIP-seq, RNA-seq, Transcription factor, Hulless barley, Lowly methylated regions, TFBS; abiotic stress

## Abstract

**Background:**

The cis-regulatory element became increasingly important for resistance breeding. There were many DNA variations identified by resequencing. To investigate the links between the DNA variations and cis-regulatory element was the fundamental work. DNA variations in cis-regulatory elements caused phenotype variations in general.

**Results:**

We used WGBS, ChIP-seq and RNA-seq technology to decipher the regulatory element landscape from eight hulless barley varieties under four kinds of abiotic stresses. We discovered 231,440 lowly methylated regions (LMRs) from the methylome data of eight varieties. The LMRs mainly distributed in the intergenic regions. A total of 97,909 enhancer-gene pairs were identified from the correlation analysis between methylation degree and expression level. A lot of enriched motifs were recognized from the tolerant-specific LMRs. The key transcription factors were screened out and the transcription factor regulatory network was inferred from the enhancer-gene pairs data for drought stress. The NAC transcription factor was predicted to target to TCP, bHLH, bZIP transcription factor genes. We concluded that the H3K27me3 modification regions overlapped with the LMRs more than the H3K4me3. The variation of single nucleotide polymorphism was more abundant in LMRs than the remain regions of the genome.

**Conclusions:**

Epigenetic regulation is an important mechanism for organisms to adapt to complex environments. Through the study of DNA methylation and histone modification, we found that many changes had taken place in enhancers and transcription factors in the abiotic stress of hulless barley. For example, transcription factors including NAC may play an important role. This enriched the molecular basis of highland barley stress response.

**Supplementary Information:**

The online version contains supplementary material available at 10.1186/s12864-022-09070-x.

## Background

Barley (*Hordeum vulgare L.*) is one of the major cereals grown worldwide and among the oldest of domesticated crops [1]. Hulless barley has been the staple food of Tibetans in China for thousands of years [2]. The genome and variome of hulless barley have been examined in previous studies [3–5]. Thus far, details of the hulless barley regulome have not yet been elucidated. Projects similar to the Encyclopedia of DNA Elements (ENCODE) have emerged, with the goal of annotating the noncoding, functional genome of a given species by generating spatiotemporal maps of chromatin accessibility, TF occupancy, protein and DNA modifications, and gene expression [6, 7]. The availability of a hulless barley ENCODE would help facilitate hypothesis generation, cross-species comparisons, genome annotation, and understanding of epigenomic functions throughout plant evolution [8].

DNA methylation has been extensively studied as an epigenetic marker in mammals [9] and plants [10]. Epigenetic markers are involved in the activities of genes and transposon elements [11]. Recent studies indicate that DNA methylation can identify transcriptional enhancers, including lowly methylated regions (LMRs) [12]. It has been suggested that LMRs, non-CpG island loci that usually contains transcription factor binding sites (TFBS), act as regulatory elements that define cellular identity. This notion is further supported by the identification of LMRs—the localized CpG-poor distal regulatory regions exhibiting under average methylation of 30%, DNase I hypersensitivity, and the presence of enhancer chromatin marks—that tend to be occupied by cell-type-specific TFs [12].

The modification of histones influences the local structure of chromatin by altering its association with nucleosomal DNA or due to the recruitment of chromatin remodeling complexes [13, 14]. Dynamic shifts in the chromatin structure affect gene regulation by modifying the availability of cis-regulatory elements (CREs), which bind with combinations of TFs in a spatiotemporal manner [15]. Genetic variation of CREs contributing to alter the tissue specificity of regulatory factors can be used to develop improved phenotypes [16]. CREs can reside within the core or proximal promoters of genes and distal to their target genes, such as in enhancer sequences that influence gene expression at long range [15].

Histone modifications are linked to changes in chromatin structure. H3K4me3 marks TSS(Transcription Start Site) and promotes gene expression via recruitment of the NURF(Nucleosome Remodeling Factor) complex for chromatin remodeling [17, 18]. H3K27me3 recruits PRC1, which contributes to chromatin compaction and impedes gene expression [19, 20]. The H3K4me3 and H3K27me3 histone modifications have been broadly utilized in research on chromatin regulation [21, 22].

In our study, lowly methylated regions (LMRs) were detected using methylome data. Using ChIP-seq technology, the different histone modifications (H3K4me3 and H3K27me3) were detected in the LMRs of tolerant and susceptive varieties subjected to stress. The aim of this approach was to characterize 1) the regulatory landscape of the hulless barley genome; 2) the enhancers that are differentially modified when comparing tolerant and susceptive varieties under stress; 3) the links between the enhancers and target genes; and 4) the gene regulatory network involved in the response to stress.

## Results

### Genome-wide identification of LMRs in hulless barley

**Fig. 1:**
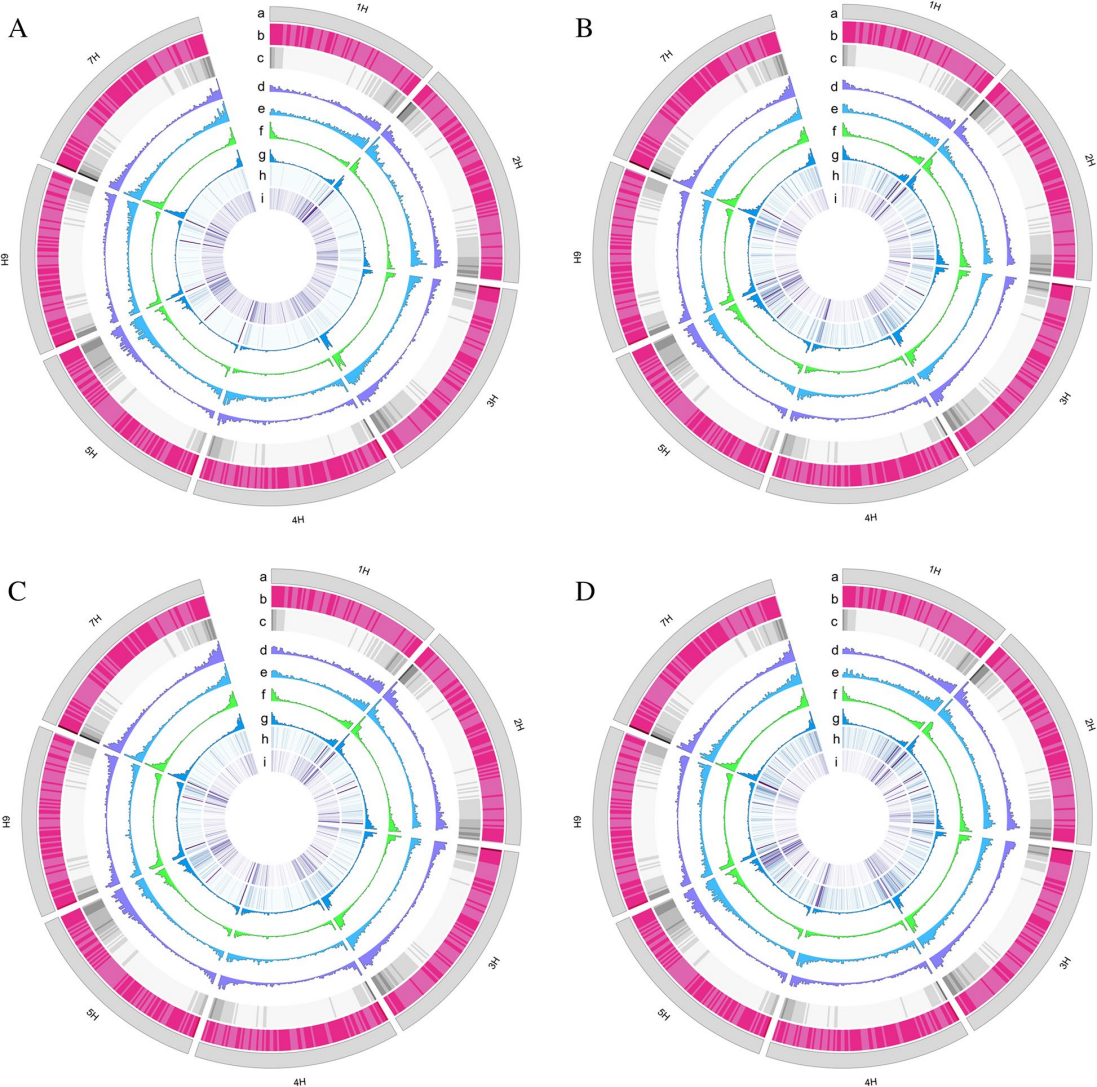
The genome-wide distribution of LMRs and histone modification. **A** The genome-wide distribution of LMRs and histone modification for XL and DQ. **B** The genome-wide distribution of LMRs and histone modification.for NC3 and NC6. **C** The genome-wide distribution of LMRs and histone modification for Z0119 and Z0226. **D** The genome-wide distribution of LMRs and histone modification for BQ 3 and GND 7. a- Chromosome ideograms, b- Repeat elements, c—Gene density, d-LMRs density from XL,NC,Z0119,BQ3, respectively e- LMRs density from DQ,NC6,Z0226,GND7,respectively;f-H3K27me3 peaks density from XL,NC,Z0119,BQ3, respectively g- H3K27me3 peaks density from DQ,NC6,Z0226,GND7,respectively, h- Density of gene expression from RNA-seq in DQ,NC6,Z0226,GND7,respectively i- Density of gene expression from RNA-seq in DQ,NC6,Z0226,GND7,respectively

To investigate the relevant regulatory elements in hulless barley under abiotic stress, we examined eight whole-genome bisulfite sequencing (WGBS) libraries (see Methods) for the genome-wide identification of LMRs that are correlated with enhancers. The abiotic stresses included in our study were drought, salinity, cold, and low nitrogen. The libraries were sequenced using Illumina Hiseq X Ten. A total of 5.1 billion 150 bp paired-end reads were generated from sodium bisulfite-treated DNA with a 99% bisulfite conversion rate for each library. On average, about 637 million sequencing reads with an average of 26 × coverage were aligned to the hulless barley reference genome for each sample (Table 1). We identified 231,440 LMRs in total and 73,249 LMRs on average for each sample. The LMRs distribution of eight hulless barley varieties under four stress conditions was consistent, which the LMRs mainly distributed in the terminal of the chromosomes at a frequency similar to the distribution of gene density (Fig. 1).

**Table1:** The sequencing results from BS-seq

Sample	RawReads	CleanReads	CleanBases	Coverage
DQ	594,788,152	590,823,228	83,104,198,973	24
XL	533,428,824	530,497,464	74,392,650,272	21
BQ3	756,520,682	748,641,934	105,687,219,276	30
GND7	737,454,714	726,820,566	100,823,741,986	29
NC3	646,283,200	641,575,332	91,217,851,918	26
NC6	681,142,904	676,647,870	96,284,006,901	28
Z0119	606,827,696	604,717,660	86,834,819,607	25
Z0226	580,123,472	577,851,246	82,416,533,291	24
Total	5,136,569,644	5,097,575,300	720,761,022,224	207
Average	642,071,206	637,196,913	90,095,127,778	26

### Histone modification in LMRs

**Fig. 2:**
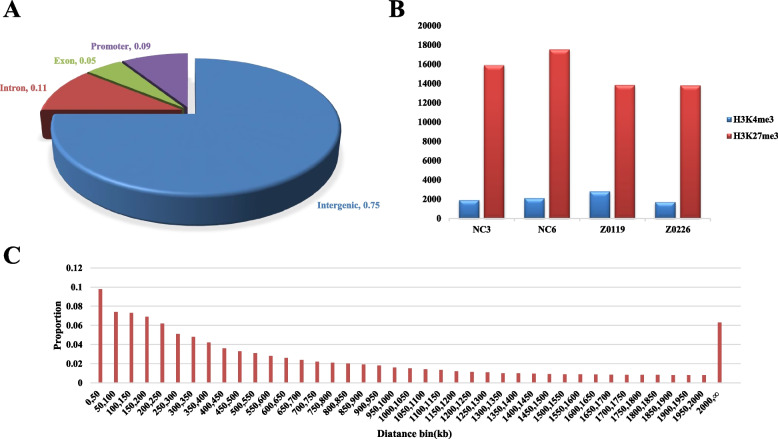
LMRs and enhancer-gene interaction pairs. A The distribution character of LMRs in the genome. **B** LMRs overlaped with the peaks of H3K4me3 and H3K27me3 modification. **C** The distance between enhancer and putative target genes

To explore the location distribution of LMR on the genome, it was found that seventy-five percent of LMRs were located in the intergenic regions of the genome (Fig. 2a) In order to examine the location of LMRs relative to sites of histone modification, 2.3 billion clean ChIP-seq reads were generated from the eight samples (mean of 94 ± 19 SD million reads per sample) (Table 2). For the H3K4me3 modification, 37,000 peaks were detected on average, whereas 43,000 peaks were detected in the case of H3K27me3 modification (Table 3). A seven-fold higher number of LMRs were found to intersect with H3K27me3 peaks than with H3K4me3 peaks (Fig. 2B). The results are in agreement with LMRs in mice [23]. H3K27me3 marks proximal and distal regulatory elements [24], whereas H3K4me3 mainly appears in transcript start sites [25].

**Table 2:** The sequencing results from ChIP-seq

Sample	RawReads	CleanReads	CleanBases
In_0119	130,331,912	120,699,622	17,133,390,386
In_0226	150,631,522	135,910,486	19,191,709,878
In_BQ3	110,756,256	96,622,958	14,027,721,777
In_DQ	84,770,450	80,465,832	11,423,743,392
In_GND7	99,378,278	85,323,580	12,349,042,608
In_NC3	85,717,146	76,974,628	11,144,732,567
In_NC6	95,183,346	85,590,522	12,406,825,955
In_XL	98,977,998	93,442,800	13,212,397,069
IP_0119_H3K27ME3	120,120,702	112,937,754	15,975,609,893
IP_0119_H3K4ME3	103,402,144	95,106,898	13,445,058,497
IP_0226_H3K27ME3	138,540,060	126,498,530	17,784,286,347
IP_0226_H3K4ME3	144,379,848	131,660,326	18,556,727,187
IP_BQ3_H3K27ME3	99,171,422	87,892,364	12,712,914,671
IP_BQ3_H3K4ME3	87,163,154	75,060,996	10,758,236,629
IP_DQ_H3K27ME3	109,696,894	103,489,644	14,706,537,230
IP_DQ_H3K4ME3	88,993,448	84,378,304	11,940,270,399
IP_GND7_H3K27ME3	125,591,094	110,606,552	15,736,874,897
IP_GND7_H3K4ME3	98,072,560	85,838,950	12,342,566,744
IP_NC3_H3K27ME3	84,343,530	75,903,352	10,976,121,973
IP_NC3_H3K4ME3	80,751,230	72,863,388	10,543,634,758
IP_NC6_H3K27ME3	83,565,116	75,700,530	10,969,572,587
IP_NC6_H3K4ME3	84,149,000	75,787,454	10,950,362,320
IP_XL_H3K27ME3	91,925,772	87,490,906	12,416,299,085
IP_XL_H3K4ME3	88,750,186	84,198,968	11,895,135,311
Total	2,484,363,068	2,260,445,344	322,599,772,160

**Table 3:** The results of calling peaks for histone modification

Samples	Peak Number	
	**H3K27ME3**	**H3K4ME3**
Z0119	54,849	43,334
Z0226	75,442	60,175
BQ3	49,582	36,347
DQ	26,127	22,963
GND7	43,166	44,047
NC3	33,355	34,742
NC6	39,553	35,151
XL	18,999	17,859

It is difficult to identify the target genes of enhancers, because enhancers can work from a distance and in either orientation [26]. In order to identify target genes regulated by distal regulatory elements, we used expression data (RNA-seq) for ten upstream and downstream genes from each distal regulatory element respectively. These 20 genes were filtered according to the correlation coefficient between the LMR DNA methylation level and gene expression level. Genes that are positively regulated by the enhancers should show a significant negative correlation. Using this method, we identified 97,909 enhancer–gene pairs, with 56,867 enhancers and 23,367 target genes (Table S1). There were 746,845 TFBS in the 56,867 enhancers, 13 TFBS in an enhancer on average. Each enhancer was associated with an average of 1.7 genes, and each gene was associated with an average of 4.2 enhancers. Of these enhancer-gene pairs, the target genes of 4468 enhancer-gene pairs were transcription factors, regarding to 1116 transcription factors. To investigate the relationship between putative enhancers and linked target genes, we determined the specific distances separating the identified enhancer–gene pairs and their frequencies using window sizes of 50 kb (Fig. 2C). We found that approximately 24% of enhancer–gene pairs were within the 150 kb range, and approximately 76% spanned 200 kb or larger genomic distances, with a median distance of 394 kb. We then selected the enhancer–gene pairs where a single enhancer was linked to a single gene and determined how often the linked gene corresponded to the nearest TSS. Approximately 59% of enhancers had only one putative target gene. Only in 5% of these putative enhancer–gene pairs was the nearest TSS.

### Analysis of transcription factors under abiotic stress

**Fig. 3:**
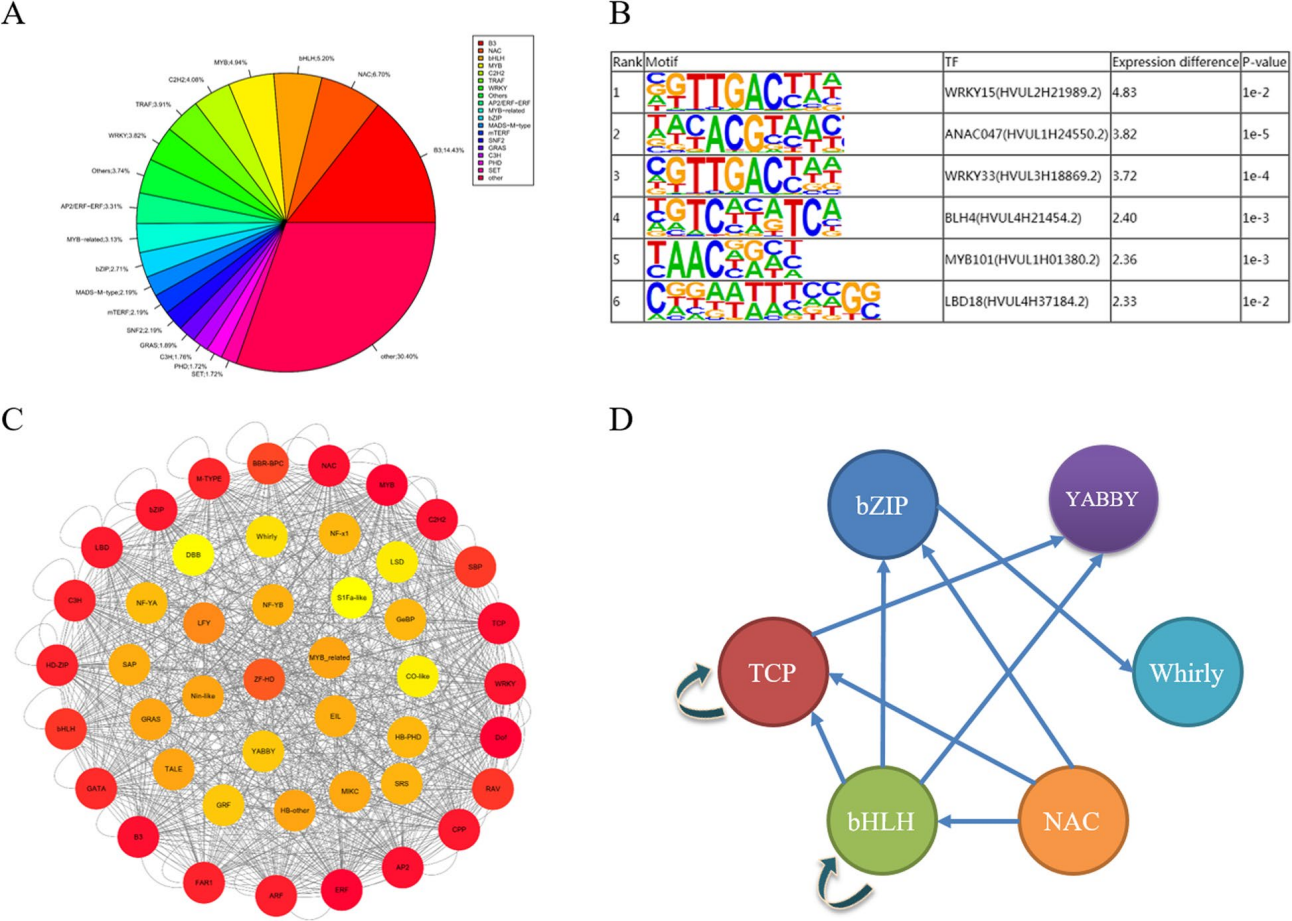
Analysis of transcription factors under abiotic stress. **A** Transcription factors identified by ITAK. **B** The drought-tolerant XL enriched motifs from the specific LMRs compared to DQ. **C** Regulatory network of of transcription factors under abiotic stress. **D**. The TF interaction network responsive to drought-stress conduction

A total of 2329 transcription factors were identified in hulless barley genome using iTAK tools, that are mainly B3,NAC,bHLH family (Table S2, Fig. 3A). The LMRs were associated with enhancers containing TF-binding sites that recruit TFs to regulate the expression of nearby genes. To identify specific TFs that may play important roles in establishing and maintaining cell fates and adapting to extreme environments, we first identified the sequence motifs that appeared to be overrepresented in the specific hypomethylated LMRs of four tolerant varieties (Fig. 3B). This identification was achieved by performing HOMER analysis on repeat-masked sequences within the LMRs. Under drought stress, 52 enriched motifs and associated transcription factors were identified, including NAC, bHLH, MYB, TCP, and bZIP. Under cold stress, 67 enriched motifs and associated transcription factors were identified, including EBF, MYB-related, NAC, AP2, bHLH, TCP, and bZIP. Under salt stress, 136 enriched motifs and associated transcription factors were identified, including TCP, MYB, NAC, and bZIP. Under low nitrogen stress, 151 enriched motifs and associated transcription factors were identified, including TCP, bZIP, HB, bHLH, MYB, NR, and AP2EREBP.

For the stress-related transcription factors, the regulatory network was constructed by Fimo, the results show that there are complex regulatory relationships among transcription factors (Fig. 3C). For the potential regulatory network under grought stressNAC transcription factor have been identified to be associated with drought stress [27]. There were 3156 significantly up-regulated genes in the XL16 sample compared to DQ, containing 181 transcription factors. There were 639 hypo-DMRs in XL16 compared to DQ, with 215 hypo-DMRs overlaped with the LMRs. Based the enhancer-gene pairs identified in the study, The target genes comprised of the transcription factors bHLH, TCP, bZIP, YABBY. Furthermore, the bHLH transciption factor could target the TCP, bZIP, YABBY transciption factors. The bHLH, TCP, bZIP transciption factors could target themselves too (Fig. 3D).

### More natural variation distributed in LMRs

To determine natural variation patters, we used variation information from previously published data sets [28]. After filtering with VCFtools, 22 million SNPs were used for subsequent analysis. The LMRs, corresponding to 75.8 Mb of sequence, were found to contain 911,187 SNPs, with 12 SNPs per kb of sequence, whereas the frequency for the whole genome is only 6 SNPs per kb on average.

## Discussion

In our study, we characterized cis-regulatory elements related to LMRs from the whole genome of hulless barley. In terms of the nature of the regulatory elements, these LMRs are thought to function as enhancers. Thus far, many species have used LMRs for identifying enhancers [29–31]. It has been shown that lowly methylated and CpG-poor regions (LMRs, ~ 30% methylation) have enhancer characteristics, such as in serving as active histone marks, promoting DNase hypersensitivity, and increasing target gene expression [32]. The links from enhancers to target genes were deciphered via correlation analysis of the relationship between methylation level and gene expression. Using this method, a total of 56,867 enhancers could be linked to 23,367 putative target genes. In the majority of cases, enhancers (59%) were linked with the expression of only one gene. We found that the putative target gene was typically not the nearest gene. In fact, the identified gene was the nearest gene in only approximately 5% of enhancer–gene pairs. We detected links under the assumption that anti-correlation between an enhancer and the expression level of a nearby gene indicates functional regulation. Further experimental studies are needed to determine, with certainty, that the enhancers regulate their putative target genes. Other assays, such as Hi-C and ChIA-PET technology can also be used to validate the indicated enhancer–gene pairs [33, 34]. Many genes could not be linked for any enhancers, the reason for this may be that the sample size was too small to allow identification of links at the required level of significance.

In 17% of cases, the LMRs overlapped with the H3K27me3 peaks, which may function as inactive enhancers [35]. Changes in the histone modification status of an enhancer region may be due to the gain or loss of site-specific transcription factors. To obtain insight into which site-specific TFs participate in response to different stresses, we examined the correspondence between stress-specific lowly H3K27-methylated enhancers and known regulatory factor recognition sequence motifs. We found associated TFs that recognize stress-specific little-H3K27-methylated enhancers, such as the NAC transcription factor [27, 36].

Inter-individual genetic variation is a major cause of phenotypic diversity. Most (88%) genome-wide association study (GWAS) loci occurred in noncoding DNA, suggesting regulatory functions [37]. In our study, we found that more SNPs were distributed in the LMRs. These SNPs are valuable resources for GWAS analysis and further functional studies.

## Conclusions

Our study primarily identified cis-regulatory elements in hulless barley via whole-genome bisulfite sequencing. A total of 231,440 lowly methylated regions were identified in our study. Through correlation with expression data, 97,909 enhancer–gene pairs were discovered for all the LMRs. The transcription factor regulatory network was inferred based on the characterization of TFBS in the CREs. The histone marks corresponding to H3K27me3 modification indicated that many LMRs were suppressed under specific abiotic stresses. The cis-regulatory elements identified in our study contained numerous single nucleotide polymorphisms in the natural population.

## Methods

### Plant materials and treatments

Eight Tibetan hulless barley (*H. vulgare* subsp. *vulgare*) varieties were used for methylome analysis in this study, which includes the drought-tolerant and susceptible varieties XL16 vs. DQ, salt-tolerant and susceptible varieties Z0119 vs. Z0226, cold-tolerant and susceptible varieties NC3 vs. NC6, and low-nitrogen -tolerant and susceptible varieties BQ3 vs. GND7. Eight hulless barley varieties were preserved in the Agricultural Research Institute, Tibet Academy of Agricultural and Animal Husbandry Sciences, Lhasa, China for many years, all the methods involving the plant and its material complied with relevant institutional, national, and international guidelines and legislation.

Drought stress was induced by 21% polyethylene glycol (PEG) 6000 solutions for 48 h. For the assessment of salinity tolerance, seedlings were stressed with 200 mM NaHCO_3_ and Na_2_CO_3_ at the two-leaf and one needle stages for 72 h, respectively.

For the assessment of cold tolerance, seedlings were grown in a growth chamber at a temperature of 4 °C for 72 h. For the assessment of low-nitrogen tolerance, sterilized seeds were germinated on moistened filter paper deficient in nitrogen in a growth chamber for 128 h. Healthy seeds were sterilized after soaking in 2% H_2_O_2_ for 40 min, rinsed in sterile water, and then germinated on moistened filter paper at 16–18 °C with 14 h light/10 h dark photoperiods and relative humidity of 80% in a growth chamber. After germination, the seedlings were grown in a growth chamber at 16 °C, relative humidity of 80%, and a photoperiod of 14 h. The roots and leaves were harvested from 10-day-old seedlings belonging to two genotypes under normal and stressful conditions. Two plants from each pot were considered biological replicates. The aforementioned tissue samples were snap-frozen in nitrogen and immediately stored at − 80 °C [38].

### DNA extraction and whole-genome bisulfite sequencing (BS-seq)

DNA was isolated using the cetyltrimethylammonium bromide (CTAB) method from hull-less barley leaves. The integrity of DNA was checked by agarose gel electrophoresis and the concentration was measured via a non-ultraviolet method [39]. Wuhan IGENEBOOK Biotechnology Co., Ltd conducted the bisulfite treatment, library construction, and sequencing. Unmethylated lambda DNA was spiked in to evaluate the conversion rate for all libraries. Finally, paired-end bisulfite-treated sample libraries were constructed and sequenced.

### Chromatin immunoprecipitation (ChIP) assay

ChIP assays were performed according to methods previously described [40] by Wuhan IGENEBOOK Biotechnology Co., Ltd (http://www.igenebook.com). Briefly, 3 g of barley leaves were washed twice in cold PBS buffer and cross-linked with 1% formaldehyde for 10 min at room temperature. They were then quenched by adding glycine (final concentration, 125 mmol/L). Afterward, samples were lysed, and chromatin was obtained following incubation on ice. The chromatin samples were sonicated to obtain soluble sheared chromatin with an average DNA length of 200–500 bp. A total of 20 μL of chromatin was stored at − 20 °C for input DNA, and 100 μL of chromatin was used for immunoprecipitation using H3K4ME3 and H3K27ME3 antibodies (9751S, CST; ab6002, Abcam). A total of 10 μg of antibody was used in the immunoprecipitation reactions at 4 °C overnight. The next day, 30 μL of protein beads were added, and the samples were further incubated for 3 h. Afterward, the beads were washed once with 20 mM Tris/HCL (pH 8.1), 50 mM NaCl, 2 mM EDTA, and 1% Triton X-100, 0.1% SDS; twice with 10 mM Tris/HCL (pH 8.1), 250 mM LiCl, 1 mM EDTA, 1% NP-40, 1% deoxycholic acid, and twice with 1 × TE buffer (10 mM Tris–Cl at pH 7.5. 1 mM EDTA). Bound material was then eluted from the beads in 300 μL of elution buffer (100 mM NaHCO3, 1% SDS) and treated first with RNase A (final concentration, 8 μg/mL) for 6 h at 65 °C and then with proteinase K (final concentration, 345 μg/mL) overnight at 45 °C. Immunoprecipitated DNA was used to construct sequencing libraries following the protocol provided by the I NEXTFLEX® ChIP-Seq Library Prep Kit for Illumina® Sequencing (NOVA-514120, Bioo Scientific) and sequenced on Illumina Xten using the PE 150 method.

### BS-seq data analysis

After removing low-quality reads, clean data were mapped to the reference genome using the software BitMapperBS (version 2.9), which permits an 8% mismatch per read [41]. Then, the methylation levels were calculated based on the methyl-cytosine percentage by MethGO [42]. Differentially methylated regions (DMRs) were identified using the software cgmaptools dmr tool [43]. LMR identification was conducted using the R package "MethylSeekR" (with the methylation level threshold under 20%) [44]. This package allows for identifying active regulatory regions from high-resolution WGBS methylomes and relies on the idea of transcription factor binding, which leads to a defined reduction in DNA methylation.

### ChIP-seq data analysis

The raw reads were filtered using Trimmomatic (version 0.38) [45]. Then, the clean reads were mapped to the hulless barley reference genome [3] by BWA (version 0.7.15) [46], allowing up to two mismatches. SAMtools (version 1.3.1) [47] was used to remove potential PCR duplicates, and MACS2 software (version 2.1.1.20160309) [48, 49] was used to call peaks of histone enrichment by default parameters (bandwidth, 300 bp; model fold, 5, 50; q-value, 0.05). The tags per kilobase of gene length per million mapped reads (TPM) were used to analyze the gene modification level (histone mark abundance).

### Linking the enhancer and target gene with methylation and expression changes

Each of the pupative enhancer-gene relations (the closest 10 upstream genes and the closest 10 downstream genes) was tested for correlation between the methylation level of the enhancer and gene expression. The gene expression data were derived from published data [50, 51]. To select these genes, the enhancer–gene distance was defined as the distance from the enhancer to the gene transcriptional start site. The enhancer was considered to regulate the target gene when the correlation was significantly negative.

### The identification of transcription factors and TFBS

Transcription factors were detected using iTAK tools [52]. A total of 2329 transcription factors were identified in hulless barley genome (Table S2). The orthologous protein pairs between proteins of hulless barley and proteins with motifs were identified in JASPAR [53], resulting in the identification of 196 orthologous protein pairs. Furthermore, we examined TFBS in the enhancer for each putative transcription factor using the software FIMO with the parameter *P* < 0.0001 [54], which allowed the association of enhancers with transcription factors in the huless barley genome.

## Supplementary Information

**Additional file 1:** **Table S1.** Identified enhancer–gene pairs.

**Additional file 2:** **Table S2.** Genome wide transcription factor identification.

## Data Availability

The datasets generated during the current study are available in the NCBI SRA repository:https://www.ncbi.nlm.nih.gov/bioproject/PRJNA708355. The experimental materials, including NC3 (langkaziqingke), Z0119, and Z0226, were landraces collected by the Tibet Academy of Agricultural and Animal Husbandry Sciences. The materials NC6 (kunlun1), BQ3 (beiqing3), GND7 (gannongda7), XL16 (ximala16), and DQ (diqingheiyuangui) were cultivars bred by different groups in western China. The plant materials used in this study are available from the corresponding author upon request.https://www.ncbi.nlm.nih.gov/bioproject/PRJNA708355

## Abbreviations

WGBS: Whole-genome bisulfite sequencing
LMRs: Lowly methylated regions
TF: Transcription factor
TFBS: Transcription factor binding site
